# Why are not all eligible chronic myeloid leukemia patients willing to attempt tyrosine kinase inhibitor discontinuation? A Czech nationwide analysis related to the TKI stopping trial HALF

**DOI:** 10.1038/s41375-024-02215-9

**Published:** 2024-03-12

**Authors:** Daniela Žáčková, Lukáš Semerád, Edgar Faber, Hana Klamová, Lukáš Stejskal, Petra Bělohlávková, Michal Karas, Eduard Cmunt, Olga Černá, Jiřina Procházková, Petra Čičátková, Anežka Kvetková, Tomáš Horňák, Ivana Skoumalová, Dana Srbová, Cyril Šálek, David Buffa, Jaroslava Voglová, Tomáš Jurček, Adam Folta, Ivana Ježíšková, Hana Žižková, Kateřina Machová Poláková, Tomáš Papajík, Pavel Žák, Pavel Jindra, Adam Svobodník, Radka Štěpánová, Jiří Mayer

**Affiliations:** 1 Dpt. of Internal Medicine Hematology and Oncology, University Hospital Brno and Masaryk University, Brno, Czech Republic; 2 Dpt. of Hemato-oncology, University Hospital Olomouc and Palacký University, Olomouc, Czech Republic; 3 Institute of Hematology and Blood Transfusion, Prague, Czech Republic; 4 Dpt. of Hemato-oncology, University Hospital Ostrava and Ostrava University, Ostrava, Czech Republic; 5 4th Dpt. of Internal Medicine and Hematology, University Hospital Hradec Králové and Charles University, Hradec Králové, Czech Republic; 6 Dpt. of Hemato-oncology, University Hospital Plzeň and Charles University, Plzeň, Czech Republic; 7 1st Dpt. of Internal Medicine – Hematology, General University Hospital, Prague, Czech Republic; 8 Dpt. Of Hematology, University Hospital Královské Vinohrady and Charles University, Prague, Czech Republic; 9 Department of Pharmacology, Faculty of Medicine, Masaryk University, Brno, Czech Republic; 10 Central European Institute of Technology (CEITEC) Masaryk University, Brno, Czech Republic

**Keywords:** Chronic myeloid leukaemia, Quality of life

## To the Editor:

Tyrosine kinase inhibitor (TKI) therapy discontinuation with the aim of achieving treatment-free remission (TFR) is becoming more frequent, as an increasing number of chronic myeloid leukemia (CML) patients are achieving stable deep molecular response (DMR; i.e. *BCR::ABL1* transcript level ≤0.01% on the International Scale); however, many challenges remain unresolved [1]. Among others, a nonnegligible proportion of patients reported fear, anxiety, or depression both during TFR and when they had to reinitiate TKI therapy [2]. Similar negative feelings are frequently mentioned in the context of the decision to not stop TKI treatment; furthermore, a considerable proportion of patients (17–50%) have been reported to be unwilling to attempt TFR [3–10] (Supplementary Table S1). However, little is known about how many truly eligible patients are unwilling to attempt TFR, the reasons for their decision and what factors are associated with their unwillingness to discontinue long-term therapy since the reports mentioned above have substantial limitations. The surveys were often conducted in a limited number of centres [3, 6–8], focused on patients who are able to use internet tools [5, 10], focused on more educated patients who are connected to patient supportive organisations [5, 7, 9], and usually not specifically focused on patients who fulfilled the criteria for TKI discontinuation [3–6, 9, 10].

In the Czech Republic, treatment for CML patients is centralised in eight specialised centres, with comprehensive data from virtually all CML patients collected in the nationwide INFINITY registry (Tyrosine Kinase Inhibitors iN FIrst aNd followIng CML Treatment). As part of the ongoing nationwide prospective multicentre investigator-initiated phase II study HALF (NCT04147533), we implemented a gradual TKI treatment discontinuation strategy. This strategy involves a stepwise dose reduction: *half* the standard dose for the first six months (*half* a year), followed by every other day administration for the next *half* a year before complete cessation (Supplementary information and Supplementary Fig. S1). Patients eligible for the HALF trial were identified based on the main inclusion criteria (Supplementary Table S2) using the INFINITY database and were educated about the study through various channels (Supplementary information). For patients who refused to participate in the HALF trial, a complementary survey called Anti-HALF was implemented to explore the reasons for not stopping TKI treatment. The paper questionnaire, with 18 questions assessing demographic information, TKI therapy, compliance, and reasons for the decision, was offered to all eligible patients who refused to participate in the HALF trial. The methods are further detailed in the Supplementary Information. Enrolment in the HALF trial has now ended. Herein, we present the results of the Anti-HALF project while the HALF trial follow-up continues.

At the initiation of the HALF study in June 2020, 1751 live patients were registered in the INFINITY database. By the end of 2022, 246 (14%) eligible candidates had been recruited to participate in the HALF trial. Within this nationwide cohort of CML patients, 190 out of 246 patients (77.2%) were enroled in the study, while 56 out of 246 (22.8%) declined to participate. Among the nonparticipants, 45 (18.3%) opted for the Anti-HALF survey, and 11 (4.5%) refused both the survey and the study. To explore the differences between HALF and Anti-HALF patients, we compared the baseline characteristics of both cohorts (Table 1). There were no statistically significant differences regarding the TKI type, CML disease duration, last TKI treatment duration, presence of TKI-related adverse events reported by patients, or TKI dose reduction at the time of study entry. Nevertheless, when evaluating the impact of dose reduction for each TKI, more Anti-HALF patients had already undergone imatinib dose reduction (*p* = 0.022). In contrast to HALF patients, the Anti-HALF group included significantly higher proportions of female patients, elderly patients, patients with only an elementary school degree education, retired patients, disabled patients, and unemployed patients. Furthermore, the Anti-HALF group reported a longer duration of their journey to the specialised haematological centre than did the HALF group. According to multivariate analyses, factors such as female sex [OR (odds ratio) = 2.3 (1.11,4.78); *p* = 0.026], longer TKI treatment duration [OR = 1.09 (1.01,1.17); *p* = 0.025], longer travel time to the centre (more than 2 h vs. up to 30 min) [OR = 5.41 (1.56,18.76); *p* = 0.008], and lower level of education [[OR = 0.27 (0.09,0.79); *p* = 0.017] for secondary school without leaving exam vs. elementary school, [OR = 0.15 (0.05,0.44); *p* = 0.001] for secondary school with leaving exam vs. elementary school, and [OR = 0.19 (0.05,0.64); *p* = 0.008]] for university vs. elementary school were significantly associated with the decision to not stop TKI treatment during the HALF study (Table 1).

**Table 1:** Characteristics of patients enroled in the HALF study or patients who participated in the Anti-HALF survey and factors associated with the decision not to participate in the HALF study.

Parameter, *N* (%) or Median [range]	Category	Anti-HALF patients (*N* = 45)	HALF patients (*N* = 190)	*p* value
Gender	Female	29 (64.4)	89 (46.8)	0.046
	Male	16 (35.6)	101 (53.2)	
Age at the time of study entry (years)		67.5 [33–89]	61.8 [24–86]	0.034
Highest level of education	Elementary	11 (24.4)	15 (7.9)	0.023
	Secondary without leaving exam	14 (31.1)	49 (25.8)	
	Secondary with leaving exam	14 (31.1)	88 (46.3)	
	University	6 (13.3)	36 (18.9)	
	UNK	–	2 (1.1)	
Type of employment	Retired/disabled/unemployed	34 (75.6)	103 (54.2)	0.017
	Employed/Self-Employed	11 (24.4)	86 (45.3)	
	UNK	–	1 (0.5)	
Travel time to the centre/clinic	Up to 30 min	8 (17.8)	43 (22.6)	0.035
	Up to 1 h	10 (22.2)	64 (33.7)	
	Up to 2 h	18 (40.0)	71 (37.4)	
	More than 2 h	9 (20.0)	12 (6.3)	
TKI used at the study entry	Imatinib	36 (80.0)	143 (75.3)	NS
	Nilotinib	9 (20.0)	31 (16.3)	
	Dasatinib	0	16 (8.4)	
TKI dose reduction at the study entry	Yes	22 (48.9)	75 (39.5)	NS
	No	23 (51.1)	115 (60.5)	
Imatinib dose reduction at the study entry	Yes	20 (55.6)	49 (34.3)	0.022
	No	16 (44.4)	94 (65.7)	
Nilotinib dose reduction at the study entry	Yes	2 (22.2)	10 (32.3)	NS
	No	7 (77.8)	21 (67.7)	
Dasatinib dose reduction at the study entry	Yes	0	16 (100.0)	-
	No	0	0	
TKI adverse events (reported by patients)	Present	19 (42.2)	70 (36.8)	NS
	Absent	26 (57.8)	120 (63.2)	
CML disease duration before the study entry (years)		9.6 [4.3–19.6]	8.7 [4.0–26.1]	NS
Last TKI treatment duration before the study entry (years)		9.2 [4.0–19.0]	8.2 [0.75–20.3]	NS
Imatinib treatment duration before the study entry (years)		11.8 [4.0–19.0]	9.9 [1.0–20.0]	NS
Nilotinib treatment duration before the study entry (years)		7.0 [4.0–13.0]	6.3 [4.0–10.0]	NS
Dasatinib treatment duration before the study entry (years)		–	4.0 [1.0–13.0]	–
**Factors associated with the decision not to participate in the HALF study (multivariate analysis)**				
**Factor**		**Effect/Unit**	**Odds ratio (95% CI)**	***p*** **value**
Gender		Female vs. Male	2.30 (1.11–4.78)	0.026
Duration of last TKI therapy		12 months	1.09 (1.01–1.17)	0.025
Travel time to the centre		More than 2 h vs. up to 30 min	5.41 (1.56–18.76)	0.008
Highest level of education		Secondary without leaving exam vs. Elementary	0.27 (0.09–0.79)	0.017
		Secondary with leaving exam vs. Elementary	0.15 (0.05–0.44)	0.001
		University vs. Elementary	0.19 (0.05–0.64)	0.008

In multivariate analysis part, only statistically significant *p* values are presented.

*N* number, *NS* not significant, *UNK* unknown, *TKI* tyrosine kinase inhibitor, *CML* chronic myeloid leukemia, *CI* confidence interval.

**Fig. 1:**
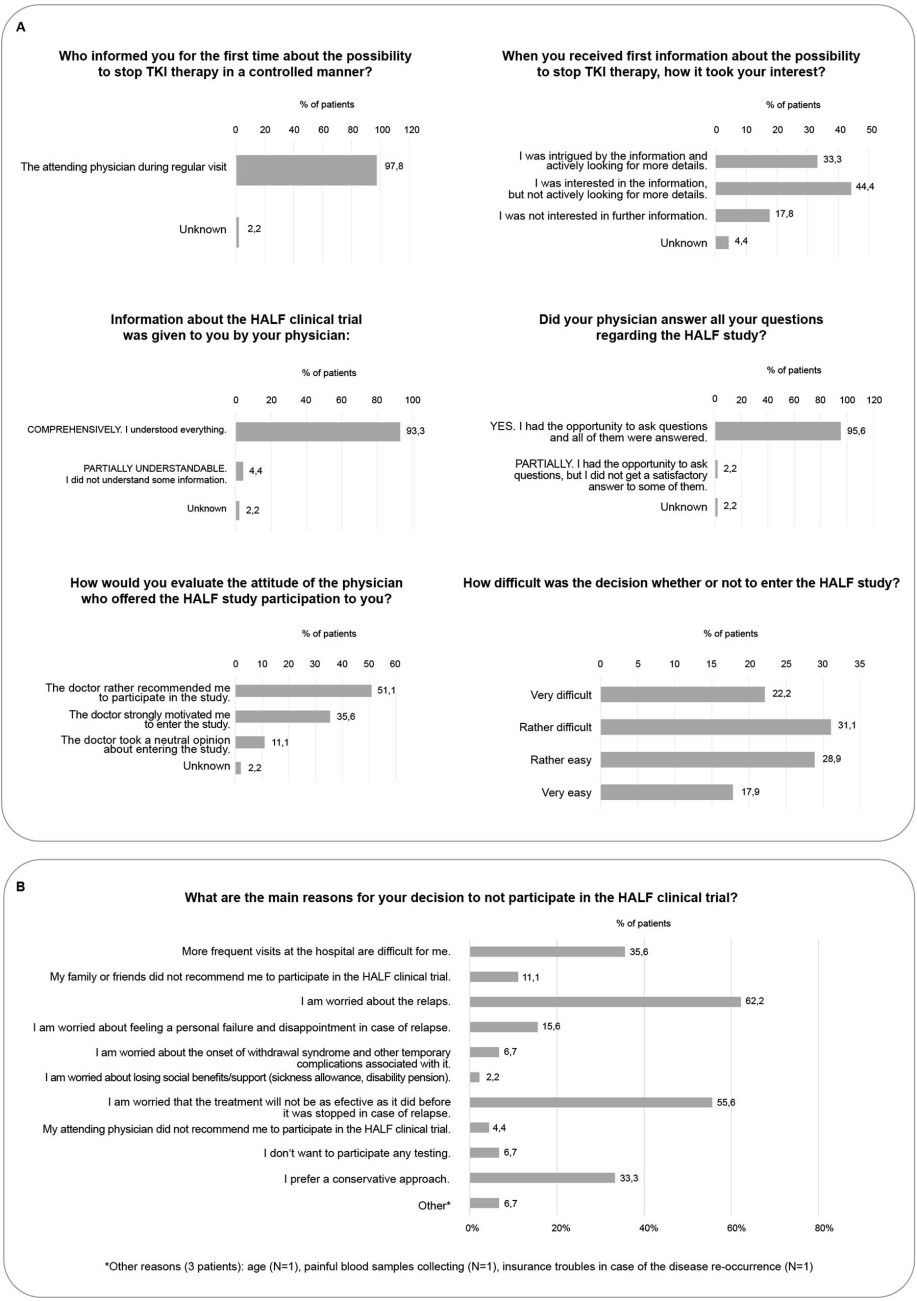
The Anti-HALF survey results. **A** Patients’ perceptions regarding treatment-free remission (TFR) proposal. **B** Main reasons for the decision to not stop tyrosine kinase inhibitors (TKI) in the frame of the HALF trial.

The analysis of perceptions regarding TKI therapy and its discontinuation was specifically focused on Anti-HALF patients (Supplementary Fig. S2 and Fig. 1). Anti-HALF patients reported minimal stress or no stress during the regular follow-up (82.2%). They perceived their TKI therapy as safe and effective (57.8%) and considered themselves very compliant (80.0%). Furthermore, more than half of these patients had never or very rarely experienced any side effects (62.2%) (Supplementary Fig. S2). Virtually all Anti-HALF patients were informed about the possibility of TKI treatment cessation by their haematologists and study investigators in one person. Most of these patients reported being highly satisfied with the information they had received (93.3%) and felt motivated to participate in the study (86.7%). The decision to enter or not enter the trial was rather difficult for them (53.3%), as they reported fear of disease recurrence (62.2%) and worries about less-effective TKI retreatment (55.6%) as the most frequent reasons for the decision not to stop TKI treatment. Additionally, patients reported some difficulties due to more frequent appointments (35.6%), a preference for a conservative approach (33.3%), and worries about feeling like a personal failure in case of disease recurrence (15.6%) (Fig. 1). Views on reasons (if any) potentially reversing Anti-HALF patients’ decisions are presented in Supplementary Fig. S3.

In our nationwide CML patient cohort, 56 out of 246 (22.8%) TFR candidates were not willing to stop TKI treatment in a controlled manner. The most frequently reported reasons for reluctance to attempt TFR in our survey align with previously published data (Supplementary Table S1) [3–10]. Villemagne Sanchez et al. emphasised, that this reluctance was often associated with a need for additional information or an incomplete understanding [7]. Flynn et al. reported patient doubts due to contradicting advice on strict medication adherence and the possibility of sudden treatment discontinuation [8]. In the same analysis, patients who refused TFR attempts were more properly informed about the generic risk of relapse after TFR than patients willing to stop [8]. Furthermore, the impact of proper perception of relapse on patient decisions was supported by an adverse relationship between increasing willingness to attempt TFR and decreasing hypothetical risk of relapse [3, 9]. Similarly, the importance of accurate information, including adequate relapse perception, was emphasised by Saglio et al. in a unique joint patient‒physician perspective on TFR [11]. In addition, the authors recommended addressing psychological aspects of TFR on a routine visit basis, in line with findings from a large Italian survey [4]. Anti-HALF patients were highly satisfied with their information and felt motivated for TFR, nevertheless, fears and worries were their most prominent emotions. Considering the aspects mentioned above and looking at the circumstances under which patients would reverse their decision not to enter the HALF trial (Supplementary Fig. S3), in some cases, a more profound and appropriate discussion might be helpful.

The results of multivariate analysis revealed significant differences between both cohorts, with greater proportion of female patients, patients treated with the last TKI longer, patients with longer travel times to the centre and patients with lower levels of education in the Anti-HALF cohort. These findings were mostly inconsistent with previously reported results (Supplementary Table S3) [3, 5–7, 9, 10]. Nevertheless, paid employment [9] and younger age [5, 6] as factors supporting willingness to attempt TFR were in line with our findings, with Anti-HALF patients being older and more frequently unemployed or retired than HALF participants. A longer TKI treatment duration was previously identified as a factor predictive for maintaining TFR [12] and for the development of TKI withdrawal syndrome [13]. Interestingly, multivariate analysis also revealed an association between longer TKI treatment duration and the refusal of TFR attempts; this association may be attributed to a stronger adherence to treatment, which was initiated during times of limited availability. Notably, some of the previous studies identified the importance of physician‒patient discussion about TFR [7] or awareness of TFR studies [9] as factors associated with the willingness to stop TKI treatment. Given the impact of factors such as educational level, age, socioeconomic factors, and long-term therapy habits on the unwillingness of Anti-HALF patients to attempt TFR, it is possible that patient‒physician discussions may need to be structured differently. However, the primary reason for rejecting TFR was the distance from the specialised centre, indicating that most eligible patients refused it for reasons other than proper and timely information.

TKI dose reduction has been increasingly shown to be safe and effective in the context of subsequent TFR attempts [14, 15]. In our study, we adopted a gradual dose reduction concept before treatment cessation to enhance patient acceptance, among other objectives. Although the results of the HALF trial have not yet been analysed, recently published data from a large Chinese survey indicated a notable preference to reduce the dose before TFR attempt in 613/817 (75%) patients *versus* 31/817 (3.8%) patients who preferred no dose reduction before stopping [10].

In conclusion, despite offering a more gradual style of TKI discontinuation and regardless of a high level of satisfaction with patient‒physician discussion, almost 1/4 of eligible patients were not willing to stop their treatment. Our analysis of factors predictive of TFR attempt refusal and the reasons for such a decision provides unique insight into patients’ perceptions regarding TKI discontinuation on a nationwide level. In very well-informed patients, logistic problems seem to be the most potent barrier.

### Supplementary information

Supplementary Information

## Data Availability

The data that support the findings of this study are available from the corresponding author upon request.
