# Supplementary material for: Why are not all eligible chronic myeloid leukemia patients willing to attempt tyrosine kinase inhibitor discontinuation? A Czech nationwide analysis related to the TKI stopping trial HALF
Source: Leukemia. 2024 Mar 12;38(4):893–7. doi: 10.1038/s41375-024-02215-9 (PMC10997522; doi:10.1038/s41375-024-02215-9)
Supplement: Supplementary file 1 — Supplementary Information [file 41375_2024_2215_MOESM1_ESM.pdf]

Supplementary Information

Why are not all eligible chronic myeloid leukemia patients willing to attempt tyrosine kinase inhibitor discontinuation? A Czech nationwide analysis related to the TKI stopping trial HALF

Žáčková Daniela[1]*, Semerád Lukáš[1]*, Faber Edgar[2], Klamová Hana[3], Stejskal Lukáš[4], Bělohlávková Petra[5], Karas Michal[6], Cmunt Eduard[7], Černá Olga[8], Procházková Jiřina[1], Čičátková Petra[1], Kvetková Anežka[1], Horňák Tomáš[1], Skoumalová Ivana[2], Srbová Dana[3], Šálek Cyril[3], Buffa David[4], Voglová Jaroslava[5], Jurček Tomáš[1], Folta Adam[1], Ježíšková Ivana[1], Žižková Hana[3], Machová Poláková Kateřina[3], Papajík Tomáš[2], Žák Pavel[5], Jindra Pavel[6], Svobodník Adam[9], Štěpánová Radka[9] and Mayer Jiří[1,10]

[1]Dpt. of Internal Medicine Hematology and Oncology, University Hospital Brno and Masaryk University, Brno, Czech Republic
[2]Dpt. of Hemato-oncology, University Hospital Olomouc and Palacký University, Olomouc, Czech Republic
[3]Institute of Hematology and Blood Transfusion, Prague, Czech Republic
[4]Dpt. of Hemato-oncology, University Hospital Ostrava and Ostrava University, Ostrava, Czech Republic
[5]4th Dpt. of Internal Medicine and Hematology, University Hospital Hradec Králové and Charles University, Czech Republic
[6]Dpt. of Hemato-oncology, University Hospital Plzeň and Charles University, Plzeň, Czech Republic
[7]1st Dpt. of Internal Medicine – Hematology, General University Hospital, Prague, Czech Republic
[8]Dpt. Of Hematology, University Hospital Královské Vinohrady and Charles University, Prague, Czech Republic
[9]Department of Pharmacology, Faculty of Medicine, Masaryk University, Brno, Czech Republic
[10]Central European Institute of Technology (CEITEC) Masaryk University, Brno, Czech Republic

List of Supplementary Tables

Supplementary Table S1. Overview of published reports on patients' willingness to attempt treatment-free remission.

Supplementary Table S2. Eligibility criteria in the HALF study.

Supplementary Table S3. Overview of factors and variables associated with patients' willingness to attempt treatment-free remission.

List of Supplementary Figures

Supplementary Figure S1. Design of the HALF trial and main inclusion criteria.

Supplementary Figure S2. Anti-HALF patient perceptions regarding TKI treatment and adherence to medication.

Supplementary Figure S3. Anti-HALF patients view the circumstances in which they would become willing to enter the HALF trial.

Supplementary Materials and Methods

The **INFINITY** (Tyrosine Kinase Inhibitors iN FIrst aNd followIng CML Treatment) **database** is a detailed noncommercial database established in 2006 that collects data from all CML patients treated in the Czech Republic using the Clinical Data Warehousing Information System (CLADE-IS) platform. All 8 centres of highly specialised haematology care have been contributing to the database in the frame of collaboration within the ***CzE*ch *L*eukemia Study Group – for *L*ife (*CELL*)** (Home - CELL - the Czech leukemia study group for life (leukemia-cell.org)).

The **HALF study** is a nationwide prospective multicentre investigator-initiated interventional phase II clinical study (EUDRACT No. 2019-003221-16, ClinicalTrials.gov NCT04147533) evaluating the efficacy and safety of TKI discontinuation after a previous two-step dose reduction in patients with chronic myeloid leukemia (CML) who have achieved deep molecular response (DMR) (for design and main inclusion criteria, see Supplementary Fig. 1, and for more study details, see https://clinicaltrials.gov/study/NCT04147533). The study was launched in June 2020, and patient recruitment was closed on June 30th, 2023. The study is still active, and the final data collection is planned for June 2026. All 8 centres for highly specialised haematology care in the Czech Republic have been participating, with Masaryk University in Brno serving as the Study Sponsor. All study examinations and procedures are detailed in the Study protocol version 1.4 from 20/6/2020, and all relevant data are collected in Case Report Forms (CRFs) in the Research Electronic Data Capture (REDCap) web application.

The **Anti-HALF survey** is a paper questionnaire containing 18 questions regarding sex; age; education; occupation; socioeconomic status; duration of patient journey from the place of residence to the centre of highly specialised haematology care; perceptions regarding TKI therapy; type of TKI currently used; adherence to medication; subjective TKI side effects and their severity; timing, quality and perceptions of the information about TKI therapy discontinuation during the HALF trial received by physician; perceptions about the decision to stop the treatment or not; and the main reasons for refusing to participate in the HALF trial. A final open-ended question asked participants about what circumstances could have changed their decision not to enter the trial. The questionnaire was offered to every HALF trial candidate refusing study participation by their treating CML specialist and HALF trial investigator in one person.

Before the HALF study launch, both eligible candidates, identified using the INFINITY database, and the HALF trial investigators were repeatedly informed about the study by multiple channels, including several national meetings, the publicly available video-interview between the study principal investigator and chair of the Czech patient supportive organisation "Diagnóza leukemie" (available from:

https://www.youtube.com/playlist?list=PLZpMSlgWJKrNxbOuMMWypgBWQJMMDFcsX) and social media involvement.
All projects described above have been approved by appropriate ethical review authorities, and recruited patients signed an informed consent form (ICF) with participation. With regard to the INFINITY database, the latter rule has been applied only to living patients since for deceased retrospectively included patients, the ICF was missing.

Statistical methods

Patient characteristics were analysed descriptively. Medians and ranges (minimum-maximum) are presented for continuous variables, and absolute and relative counts are presented for binary and categorial variables. For the comparison of variables between cohorts, the Mann–Whitney test and Fisher's exact test were used for continuous and categorical variables, respectively. To explore the factors influencing the decision to not stop TKI treatment, univariate and multivariate logistic regression models were used. All factors significant at a 10% level of significance in the univariate analysis were included in the multivariate model. For factors significant at a 5% level of significance in the multivariate model the odds ratio and its 95% CI are presented together as the p value of the Wald test.

Supplementary Table S1. Overview of published reports on patients' willingness to attempt treatment-free remission.

Reference	N (of interviewed patients)	% of patients willing to attempt TFR	% of patients refusing TFR attempt	% of pts. considering TFR attempt in some circumstances	Eligibility for TFR by ELN 2020[1]	Main reasons for TFR attempt refusal (%)	Methods
**Sanford et al., 2014[3]**	56	71%	29%	NA	No	Worries about CML relapse (80%*) and not responding to TKI retreatment (70%*)	Survey, single centre
**Breccia et al., 2015[4]**	1133	16%	49%	32%	No	Afraid of losing the results achieved thus far	Survey, multicentric
**Jiang et al., 2016[5]**	888	83%	17%	NA	No	Fears of relapse and/or TKI resistance (91%)	Survey, multicentric, distribution by PSO
**Lou et al., 2018[6]**	329	34%	39%	27%	No	Afraid of relapse (91%)	Survey, 3 centres, standardised
**Willemagne Sanchez et al., 2018[7]**	87	81%	19%	NA	Likely to be (MR4.0)	Fear of consequence of TKI discontinuation	Survey, letters/phone calls, PSO
**Flynn et al., 2019[8]**	22	50%	50%	NA	Some	Perceived risk of relapse	Survey, 3 centres, LAST study
**Tromp et al., 2021[9]**	185[¥]	76%	24%	NA	No	Fear of an aggressive disease relapse (36%*)	Survey, various channels, PSO
**Chen et al., 2023[10]**	1326	80%	20%	NA	Not specified	Fear of relapse (75%) and of poor outcome of restarting TKI (38%)	Survey, online

*Of all interviewed patients, including those who were willing to stop TKI; ¥34/185 interviewed patients who had already discontinued TKI

Abbreviations: N, number; TFR, treatment-free remission; ELN, European LeukemiaNet; NA, not applicable; TKI, tyrosine kinase inhibitor; PSO, patient supportive organisation; LAST Study, Life After Stopping Tyrosine kinase inhibitors Study; MR4.0, molecular response with 4log reduction of *BCR::ABL1* transcript levels from the baseline on International Scale, i.e., *BCR::ABL1* ≤ 0.01 (IS)

Supplementary Table S2. Eligibility criteria in the HALF study.

Inclusion criteria (Patients eligible for inclusion in this study must meet **all** the following criteria)**:**

1. **Patients with documented Philadelphia chromosome-positive and/or *BCR::ABL1*-positive CML in a documented first chronic phase, the criteria of which are as follows:**
    - < 15% blasts in peripheral blood (PB) or bone marrow (BM)
    - < 30% blasts plus promyelocytes in PB or BM
    - < 20% of basophils in PB
    - ≥ 100 x 10^9/L platelets
    - No evidence of extramedullary leukemic involvement except for hepatosplenomegaly
2. Age ≥ 18 years
3. Signed informed consent to study participation
4. Typical [e13a2 (b2a2) or e14a2 (b3a2)] or atypical quantifiable type of *BCR::ABL1* transcript on an International scale
5. TKI treatment in the first line or in the second or subsequent lines for intolerance only
6. Previous TKI treatment lasting > 4 years
7. Previous interferon-α treatment allowed with any treatment effect (intolerance/failure)
8. Deep molecular response (at least MR4.0) lasting > 2 years
9. Participants in a fertile age must agree to use prescribed contraceptive methods from entry to study until one year after the last dose of study medication:
    - Females: Proper use of a highly reliable contraceptive method, i.e. combined hormonal contraceptives (in oral, vaginal, or transdermal dosage form), gestagen hormonal contraceptives associated with ovulation inhibition (in oral or injectable dosage form), non-hormonal or hormone-releasing intrauterine device (IUD), or presence of bilateral tubular occlusion, partner vasectomy, or adherence to sexual abstinence
    - Males: Observance of sexual abstinence or use of adequate contraceptive method (i.e. condom) in the case of sexual intercourse for the period from enrolment to 1 year after the last dose of the drug

Exclusion criteria (Patients eligible for this study must not meet **any** of the following criteria)**:**

1. Patients with Philadelphia chromosome-positive and/or *BCR::ABL1*-positive CML in the second chronic phase, in the accelerated phase or blast phase at any time in the history of the disease
2. Non-quantifiable type of *BCR::ABL1* transcript on an International scale
3. TKI treatment in second or subsequent lines due to treatment failure according to ELN criteria published in 2006, 2009 or 2013

4.	Previous failure of TKI treatment according to ELN criteria published in 2006, 2009 or 2013
5.	Previous allogeneic hematopoietic stem cell transplantation
6.	Previous participation in a TKI withdrawal study with a real withdrawal history
7.	Previous discontinuation of TKI outside the study for other reasons (e.g. intolerance or pregnancy) lasting more than 9 months and/or if a treatment response was lost during less than 12 months prior to screening
8.	Life expectancy of less than 36 months due to severe concurrent disease
9.	Severe concurrent disease that could limit adherence to study protocol or study completion
10.	Pregnancy and breastfeeding
11.	Disagreement or impossibility to comply with the contraceptive measures described in point 9 of the inclusion criteria

Abbreviations: CML, chronic myeloid leukemia; TKI, tyrosine kinase inhibitor; PB, peripheral blood; BM, bone marrow; IUD, intrauterine device; ELN, the European LeukemiaNet; MR4.0, molecular response with 4log reduction of *BCR::ABL1* transcript levels from the baseline on International Scale, i.e., *BCR::ABL1* ≤ 0.01% (IS)

Supplementary Table S3. Overview of factors and variables associated with patients' willingness to attempt treatment-free remission.

Reference	N (of interviewed patients)	% of patients willing to attempt TFR	Variables tested with respect to their impact on willingness to attempt TFR	Variables confirmed (usually with multivariate analysis) with respect to their impact on willingness to attempt TFR
**Sanford et al., 2014[3]**	56	71%	Age, sex, education, annual income, insurance coverage for medications, payment concerns, adherence, side effects, CML disease duration	Sex (males) (only borderline, *p* = 0.05)
**Breccia et al., 2015[4]**	1133	16%	Not reported	Not reported
**Jiang et al., 2016[5]**	888	83%	Age, sex, education, CML disease duration, TKI therapy duration, TKI types, out-of-pocket costs, actual treatment response, TKI treatment impact on QoL	Age (younger pts.), higher out-of-pocket expense
**Lou et al., 2018[6]**	329	34%	Age, sex, education, CML disease duration, disease symptom burden, TKI types, adherence, financial difficulty, out-of-pocket cost	Age (younger pts.), CML disease duration (shorter), disease symptom burden (higher)
**Willemagne Sanchez et al., 2018[7]**	87	81%	Age, sex, CML disease duration, depth of NR, conversation with doctor about TFR, TKI side effects, treatment adherence, health-beliefs-perceived (CML-HBMQ scales*)	All CML-HBMQ scales*; the trend was also present with conversation with the doctor about TFR (*p* = 0.053)
**Flynn et al., 2019[8]**	22	50%	Not reported	Not reported
**Tromp et al., 2021[9]**	185[¥]	76%	Age, sex, education, household, paid employment, (non)academic hospital, membership in PSO, being aware of TFR studies, CML disease duration, TKI types, treatment adherence, TKI side effects, QoL	Paid employment, being aware of TFR studies
**Chen et al., 2023[10]**	1326	80%	Age, sex, education, household, marital status, disease phase at Dx, TKI Tx duration, TKI types, treatment response, TKI dose, previous TKI resistance, financial burden, poor QoL, anxiety, depression	Unmarried status, longer TKI Tx duration (> 50 months), no history of TKI resistance, poor QoL

*CML-HBMQ scales = Health Belief Model Questionnaire representing the four domains of perceived susceptibility to relapse, seriousness of relapse, benefits of stopping treatment, and barriers to stopping treatment. On average, participants who would be willing to stop TKI treatment reported lower perceived susceptibility to relapse, severity of relapse and barriers to stopping relapse. They also reported higher perceived benefits of stopping treatment; ¥34/185 interviewed patients already discontinued TKI.

Abbreviations: N, number; TFR, treatment-free remission; ELN, European LeukemiaNet; NA, not applicable; TKI, tyrosine kinase inhibitor; PSO, patient supportive organisation; LAST Study, Life After Stopping Tyrosine kinase inhibitors Study; MR4.0, molecular response with 4log reduction of *BCR::ABL1* transcript levels from the baseline on International Scale, i.e., *BCR::ABL1* ≤ 0.01 (IS); QoL, quality of life.

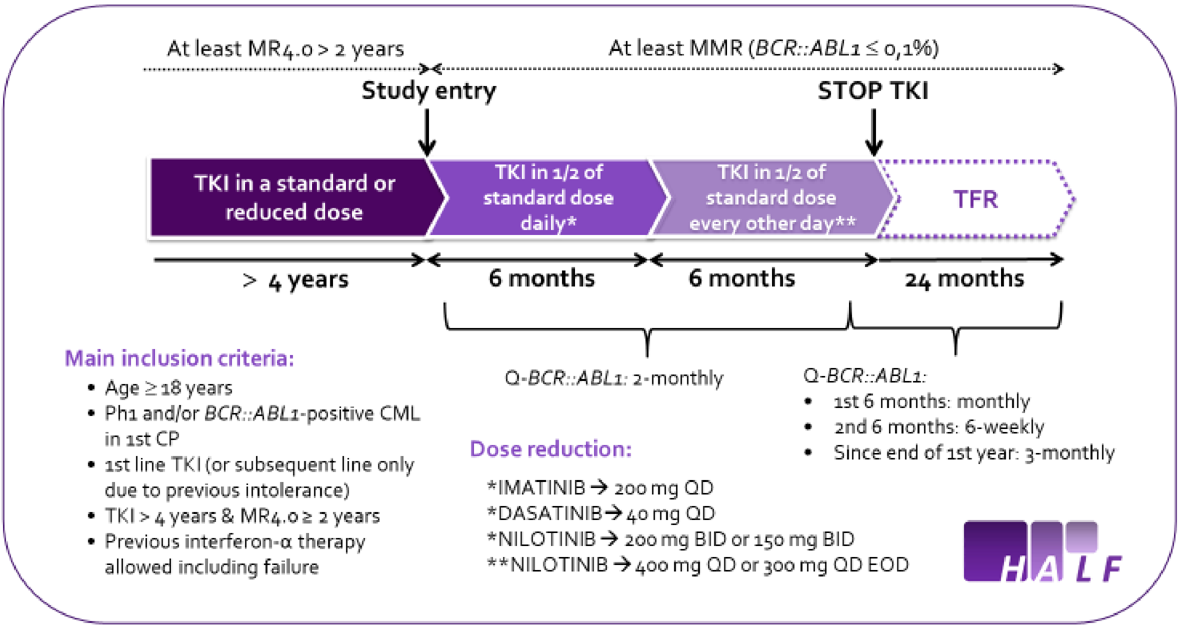

Supplementary Figure S1. Design of the HALF trial and main inclusion criteria.

Abbreviations: TKI, tyrosine kinase inhibitor; TFR, treatment-free remission; MR4.0, molecular response with 4log reduction of *BCR::ABL1* transcript levels from the baseline on International Scale, i.e., *BCR::ABL1* ≤ 0.01 (IS); MMR, major molecular response, i.e., *BCR::ABL1* ≤ 0.1 (IS); Ph1, Philadelphia (chromosome); CML, chronic myeloid leukemia; CP, chronic phase; QD, quaque die = once a day; BID, bis in die = twice a day.

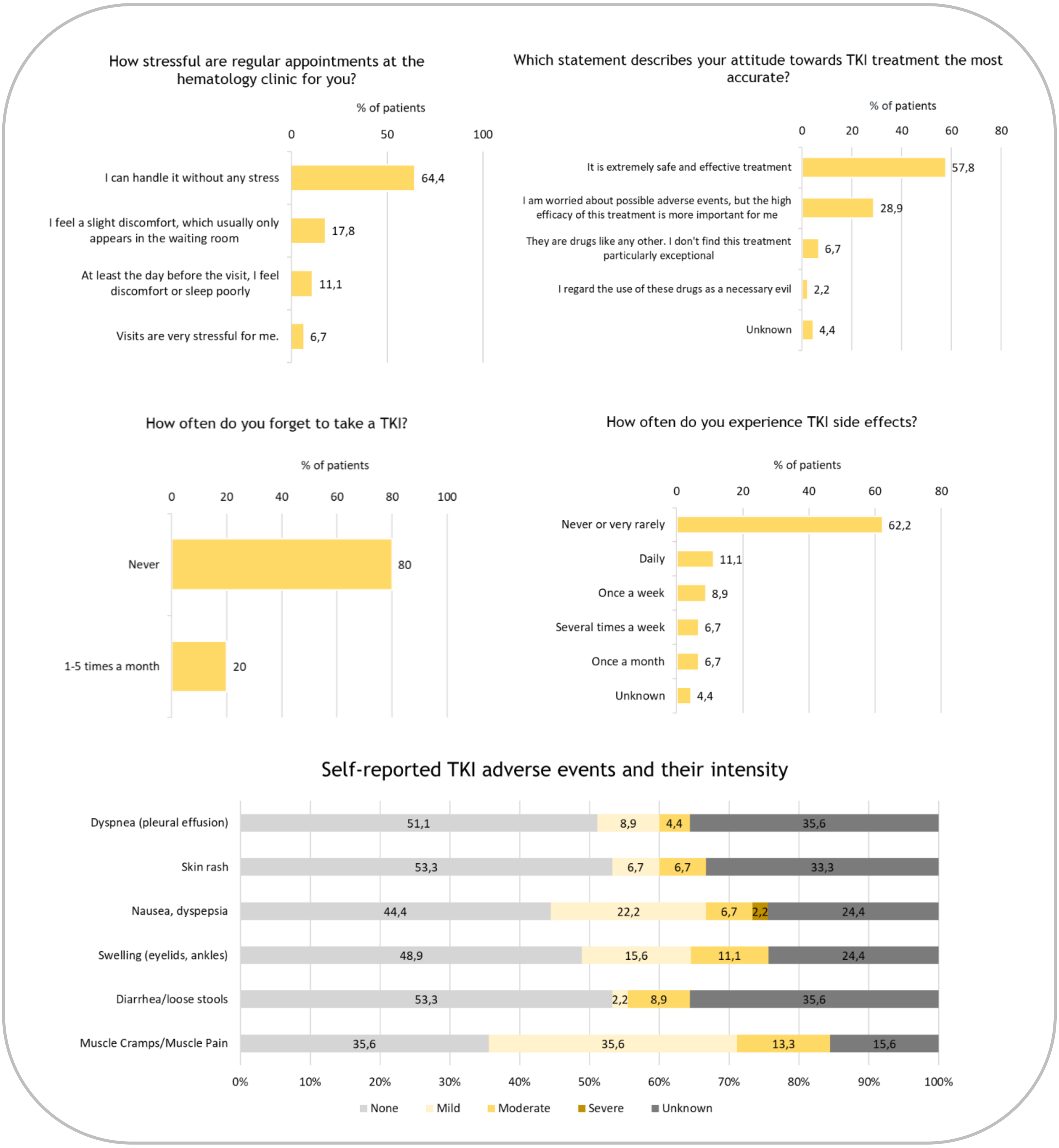

Supplementary Figure S2. Anti-HALF patient perceptions regarding TKI treatment and adherence to medication. Abbreviations: TKI, tyrosine kinase inhibitor.

Please, specify on what circumstances you would become willing to enter the HALF trial:

Patient ID	Circumstances
**AH-001-001**	None
**AH-001-004**	None
**AH-001-005**	None
**AH-001-006**	None
**AH-001-007**	Even more gradual TKI discontinuation, possibility to have some extra space in the waiting room as a study participant (not feeling safe due to COVID-19 and flu epidemy), more sufficient long-term TFR data
**AH-001-011**	Lower % of relapse risk
**AH-001-014**	100% assurance that the disease will not come back
**AH-001-019**	Less blood tests
**AH-05-001**	Shorter distance to get to the hospital
**AH-05-003**	Availability of the specialised care in the local hematology
**AH-05-004**	Shorter distance to get to the hospital
**AH-05-005**	Hospital would pay insurance if something wrong happens and there will be impossibility to continue with paid employment
**AH-06-006**	None
**AH-07-003**	None, I do not want to participate
**AH-07-006**	None, I am not willing to participate
**AH-07-010**	Living closer to the centre providing the study
**AH-08-003**	Fears of disease recurrence contributes to the worsening of pre-existing asthma and proctocolitis

Supplementary Figure S3. Anti-HALF patients view the circumstances in which they would become willing to enter the HALF trial. With patients No. AH-001-007, AH-01-014, AH-05-005, and AH-08-003, more appropriate discussion leading to better understanding might be able to change their decision.
